# Spectral tissue sensing to identify intra- and extravascular needle placement — A randomized single-blind controlled trial

**DOI:** 10.1371/journal.pone.0172662

**Published:** 2017-03-09

**Authors:** Andrea J. R. Balthasar, Geert-Jan van Geffen, Marjolein van der Voort, Gerald W. Lucassen, Stefan Roggeveen, Ivar J. Bruaset, Joergen Bruhn

**Affiliations:** 1 Department of Anesthesiology and Pain Medicine, Maastricht University Medical Center, Maastricht, The Netherlands; 2 Department of Anesthesiology, Radboud University Medical Center, Nijmegen, The Netherlands; 3 Philips Healthcare, Best, The Netherlands; 4 Department of Anesthesiology, Sint Maartenskliniek, Nijmegen, The Netherlands; Medizinische Universitat Graz, AUSTRIA

## Abstract

Safe vascular access is a prerequisite for intravenous drug admission. Discrimination between intra- and extravascular needle position is essential for procedure safety. Spectral tissue sensing (STS), based on optical spectroscopy, can provide tissue information directly from the needle tip. The primary objective of the trial was to investigate if STS can reliably discriminate intra-vascular (venous) from non-vascular punctures. In 20 healthy volunteers, a needle with an STS stylet was inserted, and measurements were performed for two intended locations: the first was subcutaneous, while the second location was randomly selected as either subcutaneous or intravenous. The needle position was assessed using ultrasound (US) and aspiration. The operators who collected the data from the spectral device were blinded to the insertion and ultrasonographic visualization procedure and the physician was blinded to the spectral data. Following offline spectral analysis, a prediction of intravascular or subcutaneous needle placement was made and compared with the “true” needle tip position as indicated by US and aspiration. Data for 19 volunteers were included in the analysis. Six out of 8 intended vascular needle placements were defined as intravascular according to US and aspiration. The remaining two intended vascular needle placements were negative for aspiration. For the other 11 final needle locations, the needle was clearly subcutaneous according to US examination and no blood was aspirated. The Mann-Whitney U test yielded a p-value of 0.012 for the between-group comparison. The differences between extra- and intravascular were in the within-group comparison computed with the Wilcoxon signed-rank test was a p-value of 0.022. In conclusion, STS is a promising method for discriminating between intravascular and extravascular needle placement. The information provided by this method may complement current methods for detecting an intravascular needle position.

## Introduction

Safe vascular access is a prerequisite for intravenous drug admission. The opposite is true during locoregional anesthesia. In this case, local anesthetics should not be injected into a blood vessel but spread around the target nerve. Inadvertent vascular injections represent rare but serious complications. Several case reports have been published since the 1940s [1]. The inadvertent injection of drugs into blood vessels can contribute to considerable morbidity [2] and, therefore, precautions should be taken to reduce the risk of intravascular injection of local anesthetics. These precautions include aspiration before injection, administration of epidural test doses of local anesthetics, and incremental administration of small amounts of drugs. Imaging techniques such as ultrasonography (US), radiography or angiography are powerful tools to choose the correct injection point and may diminish, but do not exclude, the risk of inadvertent intravascular injections [3–6]. In the case of intended venous injection, subcutaneous or inadvertent intra-arterial injections have to be avoided [7, 8].

Spectral tissue sensing (STS) is a new technique with the potential to provide information on the composition of the tissue in front of the needle tip. The technique is complementary to current imaging, aspiration and needling techniques and may be a useful tool to document and avoid inadvertent intravascular injection or to confirm an intravascular needle position. The optical method that forms the basis of the spectral tissue sensing technology is diffuse reflectance spectroscopy (DRS). The technique makes use of the phenomenon that if light is delivered to tissue, it is scattered and absorbed. The extent of scattering and absorption is determined by the composition of the tissue. For example, visible and near-infrared light are absorbed by hemoglobin, water, and lipids present in biological tissue. These substances show distinct absorption features [9–12]. Well-established techniques that make use of this phenomenon are pulse oximetry and real-time gas analysis [13, 14]. The development of fiber-optic sensing technology has enabled DRS measurements directly via the tip of a needle [15–17]. Needle stylets with DRS technology contained within standard needles allow for real-time spectral tissue sensing (STS) [18]. The STS concept used in this study has been tested in various other systems: in excised tissues, in vivo in pigs, and in vivo in humans [9, 10, 19, 20]. The theoretical advantage of STS compared to DSA and ultrasound is that STS provides real-time information on the tissue in front of the needle. Recently, Balthasar et al. demonstrated in a case series that STS was able to identify intravascular needle placement in 100% of cases compared to fluoroscopy and aspiration during invasive lumbar pain procedures [9]. In that case report, intravascular needle placement was inadvertent and STS detected the intravascular placement in off-line analysis of the data after the procedures. The goal of the current study was to investigate the potential of the STS method to discriminate intentional intravascular and non-vascular needle placement, by comparison with US and aspiration.

## Methods

### Study design

The study was conducted at the Radboud University Nijmegen Medical Center in the period of 30^th^ of June and 5^th^ of July 2011. Since there was no follow up, the data collection was completed and the study ended on the 5^th^ of July. After approval by the institutional review board (CMO Regio Arnhem-Nijmegen, Dr. F.Th.M. Huysmans, 29^th^ of June 2011) and written informed consent, 20 healthy adult volunteers participated (S1 File: Trial protocol and S2 File: Letter ethic comitee). Exclusion criteria were: pregnancy, photodynamic therapy, inability to give informed consent, category 2 and higher of the ASA physical status classification system.

The primary objective of the trial was to investigate if STS can reliably discriminate intra-vascular (venous) from non-vascular punctures.

**Fig 1:**
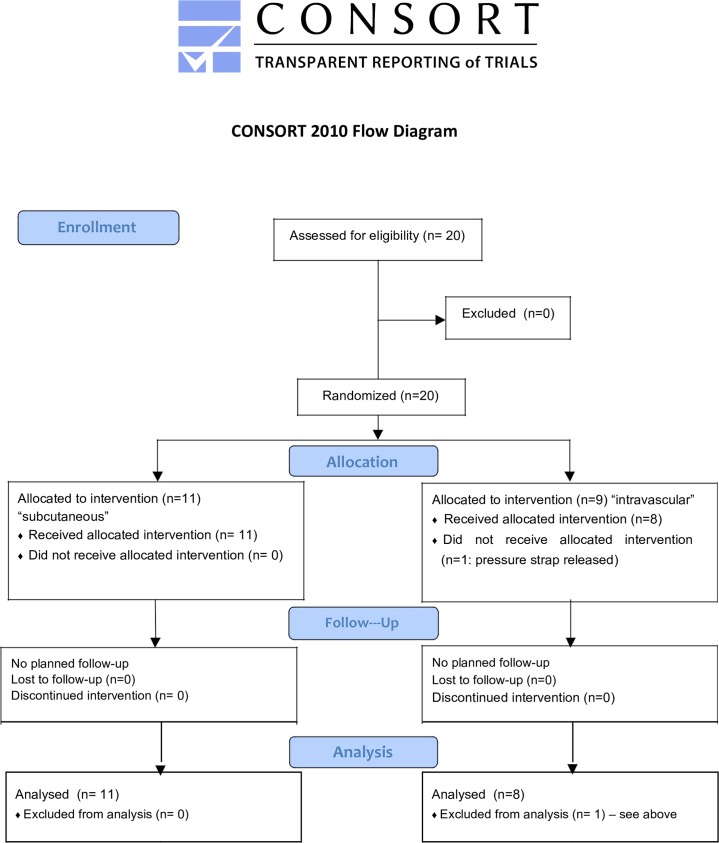
Consort flowchart of the study.

Data were obtained at one time at two locations per volunteer. The first location in all 20 volunteers was subcutaneous needle placement, while second location was determined randomly. A computerized randomization scheme was used to determine needle placement for the second measurement either intravascularly (n = 10) or in subcutaneous tissue (n = 10). The operators who collected the data from the spectral device were blinded to the insertion and ultrasonographic visualization procedure and the physician was blinded to the spectral data. The obtained reflectance spectra were analyzed offline by an investigator blinded to the procedure. No follow up was planned (see also consort flow chart, Fig 1 and consort checklist as supporting information).

According to the results of the spectral analysis, intravascular versus subcutaneous needle placement was predicted. The prediction by the STS method was compared with the “true” needle tip position as indicated by the aspiration of blood and an assessment by the physician during the procedure using out-of-plane (short-axis) ultrasound (US) guidance. A positive intravascular location was defined as a positive record made by the physician using US imaging in combination with aspiration of blood. We choose to combine both US imaging and aspiration, because aspiration alone has a sensitivity of only 25–47% to identify an intravascular needle position [21]. Ultrasound is highly dependent on the experience and interpretation of the operator and can lead to false positive as well as negative results [5]. S3 File shows the consort check list of the study.

### Study objective

The objective of this observational study is to investigate if the optical tissue stylet technology can reliably discriminate intra-vascular (venous) from non-vascular punctures.

### Study registration

This was very early phase research, sponsored by industry. Following the rules in the Netherlands for performing clinical research, the study was registered with the Dutch “Centrale Commissie Mensgebonden Onderzoek CCMO)” before enrolment was started, (see https://www.toetsingonline.nl/to/ccmo_search.nsf/Searchform?OpenForm, search term “optical tissue stylet” Identifier: NL36528.091.11). The study was internationally registered after patient enrollment: Registry Url: http://www.isrctn.com/ISRCTN15608308 Identifier: ISRCTN15608308.

### Device description

**Fig 2:**
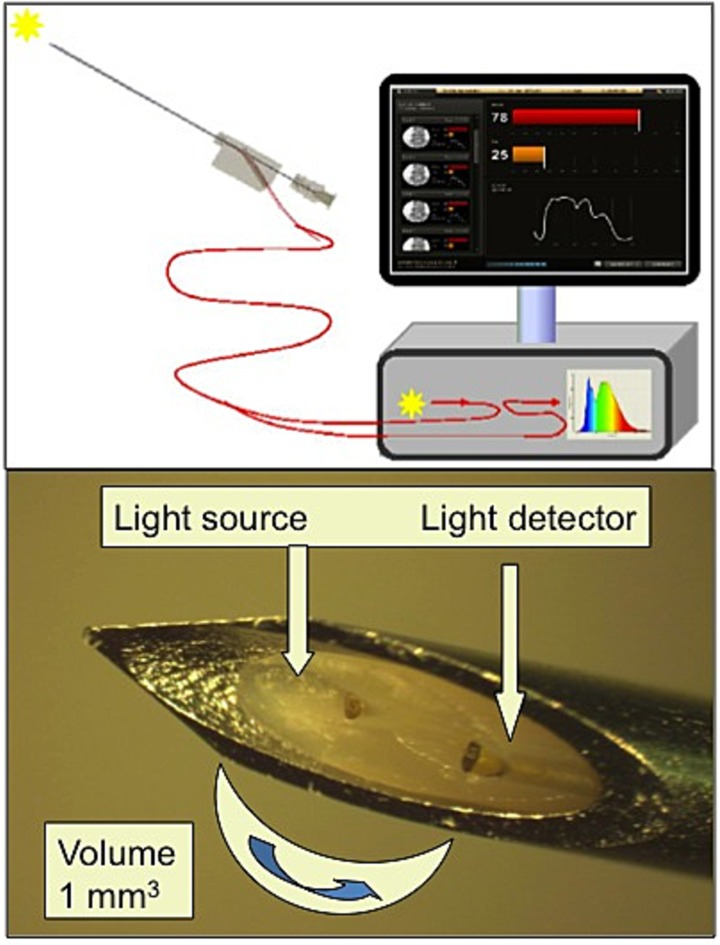
Picture of the console and needle tip. Picture of the console (upper part) and needle tip with optical stylet (lower part). The drawing demonstrates the needle, which is connected to the console. The console is just a drawing and not an accurate image of the real one. The picture of the needle tip shows the two fibers and indicates the measuring volume.

The STS system consists of two parts: an STS stylet and an optical console (Fig 2). The optical console contains a broadband (white) light source, two spectrometers and a computer board used to control the light source and data acquisition. The disposable sterile 20-G needles including the optical stylet are Conformité Européenne (CE) marked (InVivo, Schwerin, Germany). The stylet contains two 100 μm core fibers that are connected to a tungsten halogen broadband light source (500–1600 nm) and two spectrometers that receive the reflected light. A laptop computer is connected to control the light source, spectrometers and data acquisition. Details of the technical set up have been described elsewhere [9].

### Study procedures

After randomization, an experienced anesthesiologist performed all of the intravenous or subcutaneous needle insertions on the dorsal side of the hand or anterior forearm after local disinfection. During the entire procedure, a pressure strap was used on the forearm. Ultrasound imaging (SonoSite, MicroMaxs Turbo, linear transducer HFL 38 mm, 13–6 MHz) was used to guide all insertions, with the needle visualized out-of-plane (short axis). During needle insertion, spectral data were obtained twice for each volunteer: once subcutaneously and once at the intended location (subcutaneous or intravenous). Selection of the vein was done by the physician according to normal routine, preferably on the dorsal side of the hand. The needle was inserted with depth of at least 2 mm. STS has a sensing volume of approximately 1 mm^3^. No light from the STS system was visible through the skin. US images were obtained at the final needle location. At the end of the procedure, the optical stylet was removed from the needle and aspiration was performed. To prevent dislocation of the needle tip during removal of the stylet, the tip was monitored continuously on the US screen during removal. As already pointed out, aspiration of blood in conjunction with a positive US image was used as a proof of an intravascular needle position. Absence of blood aspiration in conjunction with a negative US image was used to indicate a non-vascular needle position.

Only data that were collected during procedures according to this protocol (US images, aspiration and pressure strap during entire procedure) were included in the analysis.

### Spectral analysis

Preprocessing of the spectral data (wavelength calibration, correction for instrument response and background signal, such as surrounding light) was performed in real time. After the clinical procedure, further analysis was performed using a custom-made program utilizing the software package Matlab (Mathworks, Natick, USA).

For each obtained spectrum, a “similarity” parameter was calculated, which indicates the similarity of the obtained spectrum, S_target_, with a spectrum collected from a venous blood sample, S_blood_. The blood similarity parameter is given by: *B* = 1/|<*Starget*>–<*Sblood*>|, where “< >” denotes the average of the spectral intensities at four wavelengths: 529, 545, 570, and 584 nm. A high value of the blood similarity parameter thus corresponds to high spectral similarity to blood. Because of the prominent spectral absorption from hemoglobin at these wavelengths, the blood similarity parameter provides high contrast for transition into a blood vessel. The approach followed here is similar to the analysis performed in an earlier study [9].

### Sample size calculation

We planned a study of a continuous response variable (namely the blood similarity parameter) from independent control and experimental subjects with one control per experimental subject. The sample size was determined based on the best available data at the time of the preparation of the study protocol, which were data on total hemoglobin estimates (HbT) from spectral fitting obtained in a previous study [11].

In that study, 21 “not in blood vessel” spectral measurements resulted in mean and standard deviation values for HbT of 0.0292 +/- 0.0161 and 10 “in blood vessel” spectral measurements resulted in mean and standard deviation values for HbT of 0.453 +/- 0.156. These numbers were entered into a non-parametric Mann-Whitney test (unpaired t-test) using GraphPad StatMate 2.00 and GraphPad Instat software (www.graphpad.com/statmate/statmate.htm).

From this pilot data, it can be assumed that a detectable difference between the means of HbT of 0.115 is obtained for a sample size n = 10 at a significance level of p = 0.05 and a power of 0.8. Therefore, 10 subjects with one control per experimental subject and 10 control subjects were ample.

### Statistical analysis

First, we computed blood similarity parameters for all volunteers and assessed whether a (range of) cut-off values can be determined to discriminate between values of extra- and intravascular insertion.

Next, we compared blood similarity parameters between the two groups using only the second measurements (i.e. those at the intended location) and computed the Mann-Whitney U test. We compared blood similarity parameters within the group of volunteers randomized to receive first an extravascular and then an intravascular insertion using the Wilcoxon signed-rank test, a nonparametric test for repeated measurements. A p-value of ≤0.05 was considered to indicate statistical significance. All analyses were performed using R version 3.1.3.

## Results

Data from 19 of the 20 subjects were included in the analyses. One volunteer in the vascular group (volunteer 14) had to be excluded because data acquisition was not performed according to the protocol. Thus, 38 datasets were obtained in 19 volunteers, with two datasets per volunteer. The 38 sets comprised 19 subcutaneous sets from the first measurement location and 19 sets from the second measurement location, which were divided into 11 subcutaneous sets and 8 intravascular sets. In one case, although randomization asked for intravenous needle placement, but the physician performed an intentional subcutaneous placement.

**Fig 3:**
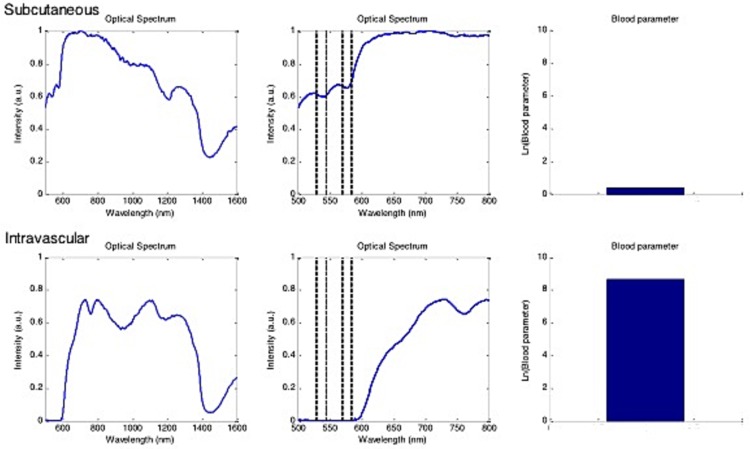
Optical spectra and the corresponding blood similarity parameters. Spectra acquired from one volunteer for subcutaneous needle (upper panes) and intravascular needle positioning (lower panes), as confirmed by positive blood aspiration. Left: full spectra, indicating the intensity of light received by the stylet (linear arbitrary units, a.u.) as a function of the wavelength (nanometers, nm). Center: an enlarged image of the spectrum that is used to determine the blood similarity parameter B (dashed lines indicate the wavelengths that are used for the calculation). Right: Ln (natural logarithm) of the blood similarity parameter as calculated for these two acquisitions.

In Fig 3, typical spectra and the corresponding calculated blood similarity parameters for a subcutaneous needle tip position and an intravascular needle tip position are shown.

**Fig 4:**
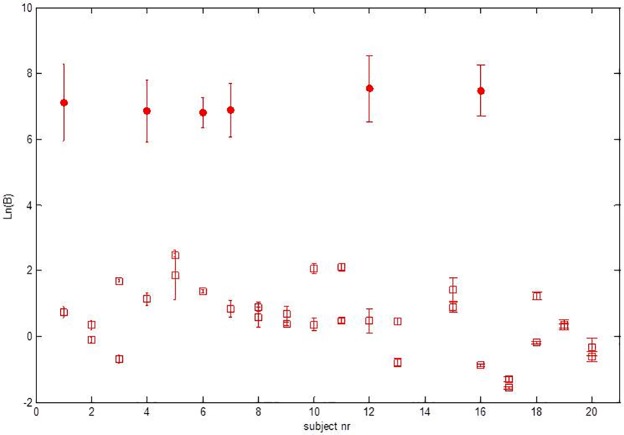
All blood similarity parameters. Overview of all blood similarity parameters (B) determined for the different measurement locations in all volunteers. Results are plotted as the average natural logarithm Ln (B) (crosses), with standard deviations determined for the set of spectra acquired at each measurement location in each subject. Volunteer 14 was excluded. Because of the considerable differences in blood similarity parameters (B) between the two groups, more details in the data are visible by plotting Ln(B) instead of B directly.

Calculated blood similarity parameters for the spectra from 19 volunteers are given in in Fig 4, where the natural logarithm of the blood similarity parameter, Ln(B), is plotted. The natural logarithm is used here to capture the large dynamic range in the blood similarity parameter B. The similarity parameter is an empirical parameter that was developed as a relative measure to compare spectra [18]. S1 Table shows the measured blood similarity parameters of al patients. The statistic analyses were performed by an independent person (Sander van Kuijk, Clinical Epidemiologist, MUMC+).

**Fig 5:**
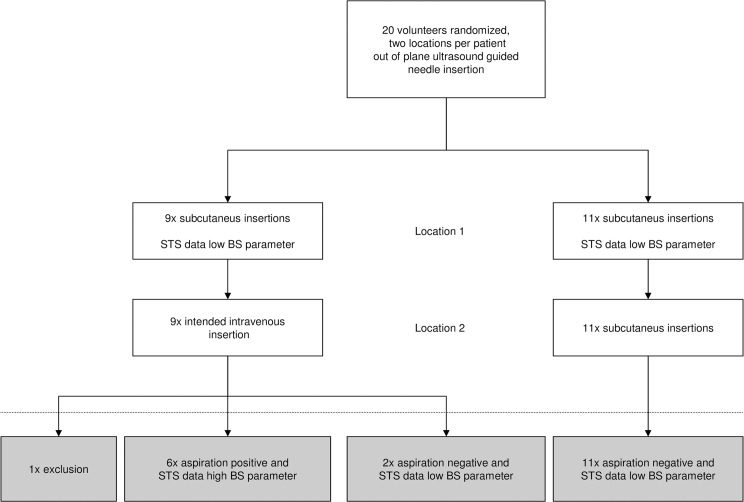
Overview study design and results. Flow diagram of the study design including the results below the dotted line. STS: spectral tissue sensing, BS: blood similarity parameter.

For 6 out of the 38 datasets, the blood similarity parameters were above the threshold, corresponding to a prediction of vascular puncture. All of these datasets corresponded with an intended vascular puncture, and were confirmed as vascular needle positions by US and the aspiration of blood. For 32 out of the 38 datasets, the blood similarity parameters were below the threshold and predicted a non-vascular needle placement. Of these 32 datasets, 19 were collected at the first, intended non-vascular measurement position. All of these were confirmed as non-vascular needle positions by US. Of the datasets where the blood similarity parameter predicted a non-vascular needle placement, 13 were collected at the second and final, measurement location. Of those 13, 11 datasets corresponded to an intended non-vascular needle position and two with an intended vascular position, confirmed by US but not by the aspiration of blood (inconclusive). Fig 5 gives an overview of the study setup and of the results.

The agreement with the predictions based on STS measurements and confirmation by blood aspiration was 100%. In comparison with US, predictions based on STS agreed with US for all intended non-vascular placements (100%, negative control) and in six out of eight cases for intended vascular placements (agreement 75%).

For the between-group comparison the natural logarithm of the mean of the extravascular and the intravascular groups was 1.36 and 8.42, respectively. The Mann-Whitney U test yielded a p-value of 0.012. The differences between extra- and intravascular were even more pronounced in the within-group comparison in those individuals that received first an extravascular insertion, followed by an intravascular insertion. The log mean values were 0.56 and 8.42 for the extra- and intravascular measurements, respectively. The p-value computed with the Wilcoxon signed-rank test was 0.022.

## Discussion

The results of the present study give some insight into the spectrum composition of intravascular versus extravascular needle placing. The ability to detect whether a needle or catheter is appropriately placed is of paramount importance for the prevention of inadvertent intravascular injection or for the confirmation of the correct, intravascular position. For instance, systemic toxicity due to inadvertent intravascular injection of local anesthetic occurs in 1 in 10000 patients after ultrasound guided axillary nerve block[3].

Balthasar et al. showed that vascular penetration events can be detected with spectral tissue sensing acquired from the tip of the needle [9]. After assessing 18 needle insertions in 10 patients undergoing sympathetic chain or communicating ramus nerve block, two inadvertent vascular penetration events were detected [9]. In the present study, we provide further evidence that an intravascular needle position can be detected by STS in human volunteers.

The results of this study show that STS can detect intravascular needle placement in 100% of cases that were positive for the aspiration of blood. There was a correspondence of 75% between STS and US. In the other 25% of cases, the anesthesiologist assumed an intravenous needle position, based on the US image. In these cases, during the insertion procedure, the needle tip positions were judged as intravenous, but after removal of the stylet, there was no aspiration of blood. The spectra obtained for these three subjects suggested a subcutaneous needle position. Intravascular needle tip positions cannot be 100% excluded with this study design. Moreover, aspiration of blood may fail to detect intravascular needle or catheter positioning. To our knowledge, detailed data for the failure rate of aspiration in this particular setting (venous puncture with aspiration and a pressure strap) is unknown. Conversely, needle tip visualization using an out-of-plane (short axis) technique is challenging and the operator may have misjudged the image [22]. It has been reported in the literature that for out-of-plane US guided procedures, the incidence of inadvertent posterior wall puncture is higher than for the in-plane approach [3, 23]. Nevertheless, there is no clear recommendation whether the out- or in-plane technique should be used during ultrasound guided vessel cannulation [24]. From a practical point of view, out-of-plane visualization of the vessel is preferred [25]. Hydrodissection is an alternative method that can be employed during US imaging to improve needle tip visualization and prevent accidental intravascular injection [26].

There are some limitations of the present study. We did not use additional in-plane imaging or the injection of saline. Practically, the stylet-containing needles used did not allow for the injection of fluid, and injection could have been performed only after removal of the stylet. In future studies, it will be important to employ disposables that allow for the injection and aspiration of fluids during the entire procedure.

Imaging techniques such as US or angiography are routinely used to confirm proper needle placement, for instance during central venous catheterization [27, 28]. An important caveat for the use of US guidance is that the needle and/or wire may not always be visualized in the vein. Confusion between the tip and shaft during US guidance of the needle can lead to inadvertent arterial cannulation [29]. The occurrence of accidental arterial cannulation is usually recognizable from the color and the pulsatile nature of the blood back flow; however, case reports suggest that this is not always true [30]. Pressure transducer monitoring and fluoroscopic guidance of the needle and catheter are considered to be reliable methods to place needles or catheters at the intended location [31]. DSA, however, uses an iodinated contrast material, which may be contraindicated and requires exposure of the patient and physician to radiation. Without the regular use of DSA during pain procedures, serious adverse events have been reported [4].

The advantages of STS are that it can provide information throughout the procedure, it does no harm to the patient or physician, it can be used to complement existing imaging modalities, STS information is less dependent on the skills and interpretation of the operator compared to DSA and ultrasound, and it does not disturb the workflow when integrated into the needle. The system used in this study was a preliminary version with a stylet. This stylet had to be removed before aspiration. It would be interesting in future studies to investigate the potential of a method to distinguish between venous and arterial blood during vascular procedures and compare with angiography and/or blood gas samples.

In conclusion, the results of this study demonstrate that STS is a promising method for discriminating between intravascular and extravascular needle placement. The information provided by this method may complement current methods for detecting and identifying an intravascular needle position and may improve patient safety.

## Supporting information

S1 File Trial protocol. (PDF)

S2 File Letter of the ethic commitee. (PDF)

S3 File Consort check list. (DOC)

S1 Table Table containing blood similarity parameters (B) of all included individuals and measurments. Individual 14 was excluded. Number 1 means first position, number 2 second position. Intended subcutaneous position (S), intended vasculair needle position (V). More data (ultrasound images, optical data and CRFs) could be found on figshare.com (project name: Spectral tissue sensing to identify intra-and extravascular needle placement).
(PDF)
