# Supplementary material for: Spectral tissue sensing to identify intra- and extravascular needle placement — A randomized single-blind controlled trial
Source: PLoS One. 2017 Mar 9;12(3):e0172662. doi: 10.1371/journal.pone.0172662 (PMC5344374; doi:10.1371/journal.pone.0172662)
Supplement: S1 File — (PDF) [file pone.0172662.s001.pdf]

PROTOCOL TITLE Optical Tissue Stylet – observational study into vascular access in humans

**Protocol ID**	
**Short title**	**OTS VA**
**Version**	**2.0**
**Date**	**June 6 2011**
**Principal investigator**	Prof. Dr. G.J. Scheffer<br>Anesthesiologist, department head<br>University Medical Centre Nijmegen (UMC St. Radboud)<br>Geert Grooteplein 10<br>6525 GA Nijmegen<br>Tel. +31 24 361 4553
**Sponsor**	Philips Electronics N.V.<br><br>Representative:<br>Stefan Roggeveen<br>Venture Manager – Photonic Needle<br>High Tech Campus 34, room 2.035, mailstop 21<br>5656 AE Eindhoven<br>Tel. +31 40 2747870
**Accredited METC**	To be submitted to Independent Review Board Nijmegen (IRBN)
**Coordinating investigator**	Not applicable
**Independent physician**	Dr. Martin Bucx, Anesthesiologist, department of Anesthesiology<br>Tel. +31 24 361 4406
**Investigators**	Dr. G.J. Van Geffen, Anesthesiologist, department of Anesthesiology, UMC St. Radboud<br>Drs. I.J. Bruaset, Anesthesiologist, UMC St. Radboud
**Study coordinator UMC St. Radboud**	Dr. G.J. Van Geffen, Anesthesiologist, department of Anesthesiology, UMC St. Radboud<br>Tel. +31 24 361 4406
**Study coordinator Philips**	Dr. M. van der Voort, Senior Scientist, Philips Research<br>Tel. +31 40 2747751
**Researchers**	Dr. G. Lucassen, Principal Scientist, Philips Research
**Investigation site**	Nijmegen University Medical Centre St. Radboud

PROTOCOL SIGNATURE SHEET

Name	Signature	Date
Sponsor's representative:<br><br>*Dr. Rob Slobbe*<br>*Director Quality and Regulatory at*<br>*Philips Corporate Technologies*		
Sponsor's representative:<br><br>*Ir. Stefan Roggeveen*<br>*Venture Manager – Photonic Needle*<br>*Philips Healthcare*		
Principal Investigator:<br><br>*Prof. Dr. G.J. Scheffer*<br>*Anesthesiologist, department head*<br>*Nijmegen University Medical Centre St. Radboud*		

TABLE OF CONTENTS

1 SUMMARY 5

2 ABBREVIATIONS AND DEFINITIONS 7

2.1 Abbreviations 7

2.2 Definitions 8

3 BACKGROUND AND JUSTIFICATION 9

3.1 Background 9

3.2 Justification 11

4 OBJECTIVES 11

4.1 Primary objective 11

5 DEVICES USED 12

5.1 Device descriptions 12

5.1.1 The optical needle stylet 12

5.1.2 The optical console 12

5.1.3 Ultrasound imaging equipment 12

5.1.4 Comments with respect to usage of the devices 13

5.2 Device disposition 13

6 TRIAL DESIGN 14

6.1 Design 14

6.2 Study population 14

6.2.1 Population characteristics 14

6.2.2 Inclusion criteria 14

6.2.3 Exclusion criteria 14

6.2.4 Sample size calculation 14

6.2.5 Withdrawal 14

6.3 Statistical considerations 15

6.3.1 Bias prevention 15

6.3.2 Data Acquisition and Statistical analysis 15

7 Methods 17

7.1 Study parameters/endpoints 17
7.1.1 Main study parameters 17
7.1.2 Other study parameters 17
7.2 General description of trial procedures 17
7.3 Monitoring and auditing 18
7.4 Early termination 18
7.5 Deviations 18
8 SAFETY REPORTING 19
8.1 Section 10 WMO event 19
8.2 Adverse and Serious adverse events 19
8.3 Follow up of 20
8.4 Data Safety monitoring board (DSMB) 20
9 DATA MANAGEMENT AND RECORD KEEPING 21
9.1 Data collection 21
9.2 Retention of data and documents 21
10 REGULATORY AND ETHICAL CONSIDERATIONS 22
10.1 Regulatory compliance 22
10.2 Amendments 22
10.3 Informed consent 22
10.4 Compensation 22
10.5 Insurance 22
11 PUBLICATION POLICY 24
APPENDIX 1 – References & bibliography 25
APPENDIX 2 – Proefpersoon informatie 27
APPENDIX 3 – Serious Adverse Event Report Form 28

1 SUMMARY

**Title**	**Optical tissue stylet – observational study into vascular access in humans**
**Purpose**	Evaluate the potential of the optical tissue stylet technology to detect needle insertion into blood vessels (veins), when easily accessible vessels with diameter comparable to the size of the veins present in the epidural space are penetrated.
**Primary objective**	The primary objective of the trial is to investigate if the optical tissue stylet technology can reliably discriminate intra-vascular (venous) from non-vascular punctures. Diffuse reflectance spectra will be acquired for these two situations, with custom-made needle stylets that contain optical fibers.
**Main study parameters**	Main study parameters are:<br>1. Successfully acquired diffuse reflectance spectra obtained in subcutaneous fat surrounding the veins in the anterior forearm, and spectra obtained with the needle tip inside veins in the anterior forearm.<br>2. Recordings of positive/negative aspiration results for the locations where the diffuse reflectance spectra have been taken.<br>3. Confirmation images by ultrasound, at the locations where the diffuse reflectance spectra have been collected.<br>4. Estimates of the diameters of the punctured veins, based on information from ultrasound imaging.<br>5. Percentages correctly identified positive and negative vessel punctures, where the identification is provided by an observer who only has access to the diffuse reflectance spectra, and is blinded to all other aspects of the procedures.
**Devices to be used**	Optical tissue stylets and needles – both will be CE marked<br>Philips ultrasound imaging equipment (CX-50) – CE marked<br>Optical console – investigational device; declaration of conformity will be signed by Philips Corporate Technologies Q&R director
**Participating parties and their roles**	Philips Healthcare – sponsor<br>Philips Research – study coordination for Philips

	Nijmegen University Medical Centre St. Radboud - investigating institution
**Study population**	Data will be collected in up to 20 healthy volunteers (category 1 of the ASA physical status classification system).<br>• Subjects who are sensitive to light (e.g. who undergo photodynamic therapy), subjects < 18 years of age, pregnant subjects, and subjects who have coagulation deficiencies will be excluded from participation
**Trial design**	This is a single-blind randomized observational study
**Trial procedures**	Volunteers will be recruited via flyers and posters. The study will encompass one visit per subject. Subjects will be randomly divided into two groups. Per group, a different needle endpoint has been defined: for one group the needle endpoint will be in the subcutaneous fat of the anterior forearm, for the other group, the needle endpoint will be inside a vein in the anterior forearm. During the visit, a needle containing an optical stylet will be inserted towards the needle endpoint, where data will be collected with the optical tissue stylet system. The position of the needle tip at the endpoint will be confirmed by ultrasound imaging and aspiration. After the measurements, the needle and optical stylet will be withdrawn and disposed of. Off-line,prediction of the needle endpoints based on the diffuse reflectance spectra will be done by a blinded observer.
**Nature and extent of the burden and risks associated with participation, benefit and group relatedness**	The procedure of inserting the needle and collecting the data will take about 5 minutes.<br>Subjects may experience some discomfort, similar to, or less than the discomfort that subjects can experience during normal blood sample collection.<br>There is no direct benefit for the group of subjects; however, the results of this investigation may in future assist the improvement of regional anesthesia and interventional pain procedures. The volunteers will receive a small but reasonable compensation for the potential discomfort.
**Milestones**	May 2011 study start; May 2011 study end

2 ABBREVIATIONS AND DEFINITIONS

2.1 Abbreviations

**AE**	**Adverse Event**
**AR**	**Adverse Reaction**
**AZM**	**Academisch Ziekenhuis Maastricht**
**CA**	**Competent Authority**
**CRF**	**Case Report Form**
**CV**	**Curriculum Vitae**
**DRS**	**Diffuse Reflectance Spectroscopy**
**IC**	**Informed Consent**
**METC**	**in Dutch: medisch ethische toetsingscommissie (Medical research ethics committee (MREC))**
**NIR**	**Near Infrared (radiation)**
**OTS**	**Optical Tissue Stylet**
**SAE**	**Serious Adverse Event**
**US**	**Ultrasound**
**UMC St. Radboud**	**Nijmegen University Medical Centre St. Radboud**
**VIS**	**Visible (radiation)**
**Wbp**	**Personal Data Protection Act (in Dutch: Wet Bescherming Persoonsgevens)**
**WMO**	**Medical Research Involving Human Subjects Act (Wet Medisch-wetenschappelijk Onderzoek met mensen**

2.2 Definitions

Definition	*Description*
Adverse Event (AE)	Any untoward medical occurrence in a subject<br>NOTE: This definition does not imply that there is a relationship between the Adverse Event and the device under investigation.
Serious Adverse Event (SAE)	An Adverse Event that<br>a) led to a death, or<br>b) led to a serious deterioration in the health of the subject that<br>resulted in a life-threatening illness or injury, or<br>resulted in permanent impairment of a body structure or a body function, or<br>required in-subject hospitalization or prolongation of existing hospitalization, or<br>resulted in medical or surgical intervention to prevent, permanent impairment to body structure or a body function, or<br>c) Led to fetal distress, fetal death or a congenital abnormality or birth defect.
Adverse Device Effect (ADE)	Any untoward and unintended response to a medical device.<br>NOTE: This definition includes any event resulting from insufficiencies or inadequacies in the instructions for use, the deployment of the device, result of user errors and applies to subjects and users.
Serious Adverse Device Effect (SADE)	An Adverse Device Effect that has resulted in any of the consequences characteristic of a Serious Adverse Event or that might have led to any of these consequences if suitable action had not been taken or intervention had not been made or if circumstances had been less opportune.
Unanticipated Adverse Device Effect (UADE)	Any serious adverse effect on health or safety or any life-threatening problem or death caused by, or associated with, a device, if that effect, problem, or death was not previously identified in nature, severity, or degree of incidence in the risk assessment or any other unanticipated serious problem associated with a device that relates to the rights, safety, or welfare of subjects.

3 BACKGROUND AND JUSTIFICATION

3.1 Background

Epidural catheter placements, as well as epidural injections are commonly used interventions providing surgical anesthesia. Adequate analgesia requires on the one hand correct identification of and needle/catheter placement inside the epidural space, and on the other hand avoidance of critical structures such as the dura mater, epidural veins, and the spinal cord. The most frequently used method for epidural space identification is the loss-of-resistance (LOR) technique. However, the method is often inaccurate, especially when used without image guidance (Curatolo, Orlando et al. 1995; Rigg, Jamrozik et al. 2002; McLeod, Roche et al. 2005). Apart from improving efficacy, imaging can play a crucial role in the prevention of side effects, by the above-mentioned avoidance of critical structures. For instance, injection of contrast under live fluoroscopy provides a reliable way to confirm absence of vascular uptake before injection of medication (Fredman, Nun et al. 1999; Araujo, More-O'Ferrall et al. 2008). Indeed, fluoroscopic guidance is strongly recommended for patients receiving epidural steroid injections (Rathmell 2005). However, fluoroscopic guidance remains impractical for routine use in surgical and obstetric anesthesia.

Also ultrasound imaging is used to guide needle placement, but with limitations as well: effects that hamper the quality of ultrasound images include bad visualization due to shadows caused by the presence of bones, and limited penetration depth (which is very relevant in for instance obese patients). As a consequence, for instance intravascular injection cannot always be prevented by ultrasound guidance (Loubert, Williams et al. 2008; VadeBoncouer, Weinberg et al. 2008; Zetlaoui, Labbe et al. 2008).

Clearly, accuracy of needle placement could be improved if information would be available that would complement the current imaging and loss-of-resistance methods. We have developed a system based on optical spectroscopy that has the potential to provide such complementary information.

The technique we employ makes use of the phenomenon that if light is delivered to tissue, it is scattered and absorbed, by amounts determined by the composition of the tissue. In particular, depending on the presence and concentrations of absorbers (also called “chromophores”) in the tissue, the light is absorbed at certain wavelengths. Prominent absorbers of visible and near-infrared light present in biological tissue are hemoglobin, water, and lipids. As these substances have distinct absorption characteristics(Schenkman, Marble et al. 1999; Bashkatov, Genina et al. 2005; Bashkatov, Genina et al. 2005; Kondepati, Heise et al. 2008), tissues that contain different amounts of these absorbers can be discriminated from each other. A well-known technique that makes use of the effect is pulse oximetry (Rithalia 1991).

The optical method that forms the basis of our system is diffuse reflectance spectroscopy (DRS). In the case of DRS, broadband light is delivered to tissue at one location, and part of the scattered light is collected at another location and analyzed spectrally (Bashkatov, Genina et al. 2005; Bashkatov, Genina et al. 2005). In recent years, the advancement of fiber optic sensing technologies has enabled DRS measurements directly from tissue at the tip of needles (Liese, Pong et al. 1985),

and needle stylets with integrated optical fibers that are compatible with standard needles have been reported. Preliminary studies have been performed into the application of needle-probe DRS for microvascular hemoglobin oxygen saturation measurement (Nezhat, Terris et al. 2004; Benaron, Parachikov et al. 2005), and breast cancer diagnosis (Bigio, Bown et al. 2000; Lubawy and Ramanujam 2004; van Veen, Amelink et al. 2005). Literature exists as well on studies into DRS for identifying peripheral nerves in dogs and rats (Abdo and Sahin 2007; Bu, Xie et al. 2008; Xie, Xiang et al. 2009) and sciatic nerves in rats (Radhakrishnan, Senapati et al. 2005; Radhakrishnan, Senapati et al. 2005). These studies suggest that peripheral nerves can be differentiated from surrounding tissues with DRS. The measurement probes used for those experiments were however incompatible with percutaneous interventions.

Recently, optical identification of the epidural space in swines was demonstrated (Ting, Tsou et al. 2010). In that study, a needle was equipped with a custom stylet with integrated optical fibers. Via the stylet, light of two different wavelengths in the visible range was delivered to and received from tissue in front of the needle tip. It was concluded from the study that the epidural space can be identified based on the reflection signatures observed for the two wavelengths. We note that in order to recognize multiple tissues or tissue chromophores, as is required when not only the epidural space has to be recognized, but the veins inside as well, diffuse reflection signatures for multiple wavelengths have to be available.

The system that we have developed aims at providing information on the tissues encountered during regional anesthesia and interventional pain procedures. It uses a disposable needle insert (“optical tissue stylet”) that enables measurement of optical spectroscopic characteristics from tissue at the tip of the needle. The stylet is equipped with optical fibers and compatible with the needles normally used for regional anesthesia and interventional pain procedures. The fibers are connected to an optical console that collects the spectra, and the system as a whole can be used in combination with image guidance.

The concept has been tested in phantoms, excised tissues, and in-*vivo* studies with swines. The results of these investigations indicate that the positioning of the needle tip can be confirmed in subcutaneous fat, muscle, blood vessels, arterial and venous blood, epidural space, and nerves, all tissues that are relevant for the physician to recognize during needle insertions (Brynolf ; Desjardins ; Desjardins ; Nachabé, Hendriks et al. 2010; Nachabé, Hendriks et al. 2010; Rathmell, Desjardins et al. 2010). Results of these preliminary studies have been promising, and justified the next step to perform a first observational study in a limited number of human subjects (van Kleef 2010). During that study, spectra were successfully collected from subcutaneous fat, muscle (both confirmed by ultrasound imaging), blood vessels (only two occurrences of accidental vessel puncture, confirmed by aspiration), and the treatment target locations close to nerves (confirmed by ultrasound imaging combined with electrical stimulation). In our opinion, proof of principle needs to be extended, in particular for vascular access and epidural access. We believe that the study as proposed in this document provides a next step towards collecting that proof for vascular access.

In case of positive results additional studies will be set up, for instance to explore the potential of the optical tissue stylet technology to detect epidural access, and accidental needle insertion into epidural veins. At a later stage, one could envision studies investigating if providing the optical tissue stylet data to the physician during a procedure results in improved procedure success rates.

3.2 Justification

We are not aware of the existence of any commercial products similar to the disposables that will be part of this study, and apart from the study that has been performed at the AZM in 2010, no comparable study has been done in humans *in-vivo*. During the study performed at the AZM, spectra were successfully collected from subcutaneous fat, muscle (both confirmed by ultrasound imaging), blood vessels (only two occurrences of accidental vessel puncture, confirmed by aspiration), and the treatment target locations close to nerves (confirmed by ultrasound imaging combined with electrical stimulation). In our opinion, proof of principle needs to be extended, in particular for vascular access and epidural access. We believe that the study as proposed in this document provides a next step towards collecting that proof for vascular access. Further, we would like to note that proof of principle has to be collected *in-vivo*, as the optical properties that form the basis for the concept can be different under different circumstances. Hence, data that have not been collected *in-vivo* will not provide sound evidence.

In particular for a vascular access study, we prefer to recruit volunteers, as we do not find it ethical to aim at unnecessary vessel puncture in patients that are scheduled for treatment. Vessel access will be studied in the forearm, in the same area where blood samples are normally collected. As no samples will be taken, the discomfort that subjects can experience will be less than during normal blood sample collection. Note as well that in this area of the forearm, vessels are located within the subcutaneous fat; within this area, the expected discomfort is less than for other areas, where vessels are located between muscles.

4 OBJECTIVES

The aim of this observational study is to evaluate the potential of the optical tissue stylet technology to detect needle insertion into blood vessels (veins).

4.1 Primary objective

The primary objective of the trial is to investigate if the optical tissue stylet technology can reliably discriminate intra-vascular (venous) from non-vascular punctures. Diffuse reflectance spectra will be acquired for these two situations, with custom-made needle stylets that contain optical fibers.

5 DEVICES USED

5.1 Device descriptions

The optical tissue stylet technology will be employed during needle insertions into the anterior forearm of volunteers. The concept contains two parts that are relevant in this test: an optical needle stylet, and an optical console.

5.1.1 The optical needle stylet

The optical needle stylet is a sterile disposable part that will be used per subject only. It is compatible with the CE-marked commercially available 20G needles. The stylet is equipped with 2 optical fibers. Through these fibers light is send into and received from the tissue. The design and production of the stylets is outsourced to Invivo, a Philips company and certified supplier. The stylets are CE marked.

5.1.2 The optical console

The fibers of the needle-stylet are connected to a console. This console contains a light source and two spectrometers that receive the light from the tissue. A laptop is connected to the console for control of the light source and spectrometers, and data acquisition. The optical console will be operated by a qualified Philips Research employee. This researcher will also take notes during the tests. The console has not been CE marked. However, we have taken all precautions to make sure that operation will be safe for the user, physician, and volunteer, and in accordance with regulations that are relevant for this feasibility test, in accordance with international safety standards. It is an investigational device, meant (and labeled) to be used during this study only. A Declaration of Conformity has been issued by the director for Quality and Regulatory at Philips Corporate Technologies. There is no electrical connection between the optical console and the optical stylet, and consequently not between the optical console and the volunteer and physician either. The device has been assessed and approved for use during this study by the “Instrumentele Dienst” of the UMC St. Radboud as well.

5.1.3 Ultrasound imaging equipment

Equipment for ultrasound imaging will be used to record confirmation images at the positions where optical spectra will be collected. Also, the diameters of the punctured vessels will be determined from US images. For this, a CE-marked Philips CX-50 ultrasound imaging system will be employed. As is normal practice, the US imaging equipment will be operated by the physician performing the treatment.

5.1.4 Comments with respect to usage of the devices

All equipment mentioned in this section will be brought in by Philips, following the procedures as set out by the “Instrumentele dienst” of the UMC St. Radboud.

The optical console will be set up and maintained by Philips, and removed from the trial site after the test. Data acquisition by the console will be controlled by a qualified Philips Research employee. Also off-line data analysis will be done by Philips Research.

The procedures will be performed by experienced anesthesiologists. When the optical tissue stylets are inserted, the handling of the needles will be very similar to usual handling of needles that contain probes, e.g. for electrical stimulation measurements or RF ablation procedures. However, to prevent surprises with respect to needle handling, all anesthesiologists will train usage of the intelligent stylets before the study.

To test the workflow during the study, before the start of the study “dry runs” will be done on test samples, to simulate and practice the procedures performed during the actual study.

5.2 Device disposition

Track should be kept of the disposables: items will be numbered with a unique code. Entering of items at the hospital will be recorded, as well as use during a certain procedure, disposal at the hospitals after use, and return to Philips in case items are not used during the study.

6 TRIAL DESIGN

6.1 Design

This single-blind randomized observational study aims at demonstrating proof of principle in humans.

6.2 Study population

6.2.1 Population characteristics

Healthy *volunteers* will be recruited at the UMC St. Radboud.

6.2.2 Inclusion criteria

- More than 18 years old
- Healthy (category 1 of the ASA physical status classification system)

6.2.3 Exclusion criteria

- Pregnancy
- Photodynamic therapy.
- Inability to give informed consent.
- Category 2 and higher of the ASA physical status classification system

6.2.4 Sample size calculation

We propose to perform measurements in 20 subjects. For all subjects, measurements will be performed at a first, non-vascular, location in the forearm. Also in all subjects measurements will be performed at a second location in the forearm. For N=10 subjects, the second location will be non-vascular as well. For the other N=10 subjects, the second location will be vascular.

6.2.5 Withdrawal

Subjects can leave the study at any time for any reason if they wish to do so without any consequences. The investigator can decide to withdraw a subject from the study for urgent medical reasons.

Voluntary participation and the way to withdraw from the study will be explained to the subjects during the intake.

The study will stop whenever the required number of subjects has been part of the study.

6.3 Statistical considerations

6.3.1 Bias prevention

Before the study start, 20 envelopes will be prepared, one for each subject, indicating if the subject will receive an intravascular or non-vascular puncture, 10 for each case. Before each puncture, the physician will randomly take an envelope out of the remaining un-opened envelopes, and perform the corresponding puncture. The information from the envelopes will be stored with the subject identification codes (see session 9.1). The operators who will collect and analyze the optical data will not know the content of the envelopes.

Likewise, data collected by the optical tissue stylet system will not be provided to the physician during the procedures, as we intend to influence the course of the procedures as little as possible.

Also the fact that several anesthesiologists will perform the punctures decreases the chance of bias to occur.

6.3.2 Data Acquisition and Statistical analysis

For each volunteer, data will be collected at 2 different locations along the needle trajectory: subcutaneously, and depending on the content of the envelope, at a second location either non-vascular, or intravascular. For each location, 10 DRS spectra will be collected.

Preprocessing of the optical spectroscopic data will be done real-time: data will be wavelength-calibrated, corrected for instrument response and background signals, and selected over a spectral wavelength range of 500-1600 nm.

After the procedures, further analysis of the data will be performed: From each spectrum, “features” will be extracted. These features are interval scaled parameters that relate to the concentrations of chromophores present in the tissues. These can be calculated using well-established algorithms (see for instance: (Zonios, Perelman et al. 1999)).

Differences between the features that are obtained from the specific locations during the punctures (subcutaneous fat, non-vascular, and intravascular) during all needle insertions will be analyzed with an ANOVA technique. Additionally, the features of two specific tissues will be compared with the Student’s t statistic.

To analyse the diagnostic performance in discriminating between non-vascular and intravascular puncture, a comparison Receiver Operating Characteristic (ROC) curve will be constructed and Areas Under the Curve (AUC) will be calculated.

Further, with the parameters extracted from the spectra, supervised classification algorithms that will predict the types of tissues will be further improved. The software package Matlab will be used for the analysis.

Finally, “similarity” parameters will be calculated, which indicate the similarity of the obtained spectra with spectra collected for subcutaneous fat and venous blood during a previous clinical study.(van Kleef 2010). These similarity parameters will be used to predict in which tissue the spectra have been collected as well.

All predictions will be compared with the “true puncture positions” as indicated by the envelopes, and. Percentages correctly identified positive and negative vessel punctures will be determined.

7 Methods

7.1 Study parameters/endpoints

7.1.1 Main study parameters

Main study parameters are:

1. Successfully acquired diffuse reflectance spectra obtained in subcutaneous fat surrounding the veins in the anterior forearm, and spectra obtained with the needle tip inside veins in the anterior forearm.
2. Recordings of positive/negative aspiration results for the locations where the diffuse reflectance spectra have been taken.
3. Confirmation images by ultrasound, at the locations where the diffuse reflectance spectra have been collected.
4. Estimates of the diameters of the punctured vessels, as determined from the ultrasound images.
5. Percentages correctly identified positive and negative vessel punctures, where the identification is provided by an observer who only has access to the diffuse reflectance spectra, and is blinded to all other aspects of the procedures.

7.1.2 Other study parameters

Other study parameters include: subject age, sex, height, weight.

7.2 General description of trial procedures

The study procedure involves the following steps:

- Recruitment will happen at the UMC St. Radboud department of anesthesiology, via posters and flyers announcing the study. Subjects can contact one of the physicians mentioned on the poster/flyers to indicate their interest to participate. If subjects indicate interest, a paper information leaflet for subjects will be handed (see also information for volunteers, in Dutch: proefpersoon informatie, see Appendix 2) After that, one of the participating physicians or a research nurse will provide information on the background and procedures to be followed during the study. From all subjects entering the study, informed consent will be obtained (see Appendix 2 for a sample informed consent form).
- Relevant subject data will be recorded on the case report form (age, sex, height, weight)
- Before each puncture, the physician will randomly take an envelope out of the remaining un-opened envelopes, and perform the corresponding puncture.
- The puncture will be performed with a 20G needle that would normally be used for anesthesia. For these needles, the stylet will be replaced by a sterile disposable optical stylet. Data will be

collected through the stylet at two instances during the procedure; for each instance 10 DRS spectra will be stored, with an acquisition time of 0.5 second each. Depending on the type of puncture (non-vascular or intravascular – as prescribed by the content of the envelope) spectra will be collected for subcutaneous fat 1 (for all subjects), subcutaneous fat 2 (for half of the subjects), or for a vascular puncture (for half of the subjects) For each instance during which optical tissue stylet data are recorded, the exact needle tip location will be confirmed by ultrasound imaging and aspiration. Also these confirmation data will be stored, and comments that relate to the location of the needle will be noted down on the CRF. The procedure involves one visit per subject.

A paper CRF will be used to record the above-mentioned relevant subject data, identification number of the used disposable OTS part, identification numbers of the files that contain the stored DRS data, and comments that relate to positioning of the needle.

7.3 Monitoring and auditing

In addition to monitoring visits, Philips Research may conduct audits at the investigation site. The purpose of an audit is to verify the adequate performance of the Clinical Investigation related activities. Regulatory bodies may also perform inspections at the investigation site.

The investigator and/or institution shall permit Philips Research and regulatory bodies direct access to source data and documents, taking into account any restrictions due to local law, to perform trial-related monitoring, audits, METC review, and regulatory inspections.

7.4 Early termination

If serious adverse effects come up that are related or can in any way be related to the study, the study will terminate. In that case, all devices (disposables and console) will be taken back by Philips Research.

In case the study is ended prematurely, the sponsor will notify the accredited METC within 15 days, including the reasons for the premature termination.

7.5 Deviations

All deviations from this protocol shall be documented and explained, regardless the reason for deviation.

8 SAFETY REPORTING

8.1 Section 10 WMO event

In accordance to section 10, subsection 1, of the WMO, the investigator will inform the subjects and the reviewing accredited METC if anything occurs, on the basis of which it appears that the disadvantages of participation may be significantly greater than was foreseen in the research proposal. The study will be suspended pending further review by the accredited METC, except insofar as suspension would jeopardise the subjects' health. The investigator will take care that all subjects are kept informed.

8.2 Adverse and Serious adverse events

All SAEs will be reported to the accredited METC that approved the protocol, according to the requirements of that METC.

At the UMC St. Radboud, SAEs will be reported to and evaluated by dr. Dr. Martin Bucx, Anesthesiologist, department of Anesthesiology, who is not involved in this study. Tel. +31 24 361 4406.

If SAEs occur, the principal investigator will inform Philips Research within 24 hours, using the form provided in APPENDIX 3 – Serious Adverse Event Report Form, either by e-mail or by fax. Additionally, the Philips Research Q&R office is notified by telephone whenever an email or fax has been sent. Phone numbers for notification: **+31 40 274 0430 or +31 40 274 8113.**

If serious adverse effects come up that are related or can in any way be related to the study, the study will terminate. In that case, all devices (disposables and console) will be taken back by Philips Research.

A summary of the communication required in case of (serious) adverse events and serious adverse device effects is given in the table below.

Category	***Report to Sponsor***	***Report to Accredited MEC***	***Report to Competent Authority***	***Report to Director Quality and Regulatory CT***
	by Principle Investigator	*by Principle Investigator*	*by Director Quality and Regulatory CT*	*By Principle Investigator*
Adverse Event (AE)	Periodic reporting to Sponsor	As part ofTrial Report	Not applicable	Periodic reporting to Sponsor
Serious Adverse Event (SAE)	Within 24 hr	As part of Accredited MEC Reporting timelines	Within 24 hr	Within 24 hr
Serious Adverse Device Effect (SADE)	Within 24 hr	Within 48 hrs	Within 10 days	Within 24 hr

Table 1: Overview of communications in case of adverse events or adverse effects

8.3 Follow up of

All adverse events will be followed up until they have abated, or until a stable situation has been reached. Depending on the event, follow up may require additional tests or medical procedures as indicated, and/or referral to the general physician or a medical specialist.

8.4 Data Safety monitoring board (DSMB)

Not applicable.

9 DATA MANAGEMENT AND RECORD KEEPING

9.1 Data collection

Subject data will be handled confidentially and an identification code will be used to link the data to the subject. The principal investigator of the UMC St. Radboud safeguards the key to the code. In particular, for this study Prof. Dr. GJ Scheffer, Dr.GJ van Geffen, and Drs. I. Bruaset of the UMC St. Radboud will have access to the personal data. The handling of personal data complies with the Dutch Personal Data Protection Act (in Dutch: De Wet Persoonsbescherming). The signed informed consent statements, the listing of the identities of the subjects, the envelopes indicating the puncture type for each volunteer, originals of the CRF's, and data collected during the study will be stored at the UMC St. Radboud.
Note: Anonymized data, as well as copies of the anonymized CRF's will be made available to Philips Research for statistical analysis and to further optimize the OTS technology.

9.2 Retention of data and documents

Subject (hospital) files will be archived according to local regulations. All documents related to the study will be retained up to at maximum 15 years after the end of the study.

At the end of this period, the investigator is to obtain permission from Philips before any documents are destroyed.

10 REGULATORY AND ETHICAL CONSIDERATIONS

10.1 Regulatory compliance

The study will be conducted according to the principles of the Declaration of Helsinki (Seoul, 2008) and in accordance with the Medical Research Involving Human Subjects Act (WMO) and other applicable guidelines, regulations and Acts.

10.2 Amendments

Amendments are changes made to the research after a favourable opinion by the accredited METC has been given. All amendments will be notified to the METC that gave a favourable opinion.

10.3 Informed consent

Recruitment will happen at the UMC St. Radboud department of anesthesiology, via posters and flyers announcing the study. Subjects can contact one of the physicians mentioned on the poster to indicate their interest to participate. If subjects indicate interest, a paper information leaflet for subjects will be handed (see also information for volunteers, see
APPENDIX 2 – Proefpersoon informatie, in Dutch) After that, one of the participating physicians or a research nurse will provide information on the background and procedures to be followed during the study. From all subjects entering the study, informed consent will be obtained (see
APPENDIX 2 – Proefpersoon informatie).
Subjects who participate in the test will not benefit from the test nor experience unacceptable additional discomfort. As described in the risk analysis report (Voort 2011) subjects will be exposed to minimal risk by taking part in the study. There is no direct benefit for the group of subjects; however, the results of this investigation may assist the improvement of regional anesthesia or interventional pain procedures.

10.4 Compensation

A small compensation (25 Euro) will be paid to the test subjects, to compensate for the discomfort that they can experience when taking part in the study. Subjects will be informed about the minimal discomfort that they can experience, similar to, or less than the discomfort that subjects can experience during normal blood sample collection.

10.5 Insurance

The sponsor has both a “General and Product Liability Insurance” and a “Clinical Trial Insurance”, in accordance with the legal requirements in the Netherlands (Article 7 WMO and the Measure regarding Compulsory Insurance for Clinical Research in Humans of 23rd June 2003). These insurances provide cover for damage to research subjects through injury or death caused by the study.

1. € 450.000,-- (i.e. four hundred and fifty thousand Euro) for death or injury for each subject who participates in the Research;
2. € 3.500.000,-- (i.e. three million five hundred thousand Euro) for death or injury for all subjects who participate in the Research;
3. € 5.000.000,-- (i.e. five million Euro) for the total damage incurred by the organisation for all damage disclosed by scientific research for the Sponsor as ‘verrichter’ in the meaning of said Act in each year of insurance coverage.

The insurances apply to the damage that becomes apparent during the study or within 4 years after the end of the study.

11 PUBLICATION POLICY

The sponsor will notify the accredited METC of the end of the study within a period of 8 weeks. The end of the study is defined as the last subject's last visit.

Within one year after the end of the study, the investigator/sponsor will submit a final study report with the results of the study, including any publications/abstracts of the study, to the accredited METC.

The Investigating Institution or Investigator must submit to Philips any abstract, manuscript, or other communication relating for review 60 days prior to submission for publication. Publications are only allowed after the Clinical investigation has been completed. Philips retains the rights to review and revise the manuscript to ensure protection of proprietary or other confidential commercial information and compliance with regulatory requirements. Publication of negative data will not be prevented. We will comply with the CCMO statement on publication policy

APPENDIX 1 – References & bibliography

Abdo, A. and M. Sahin (2007). NIR light penetration depth in the rat peripheral nerve and brain cortex, IEEE.

Araujo, M., D. More-O'Ferrall, et al. (2008). "Complications of Interventional Pain Management Procedures." Advances in Anesthesia **26**: 1-30.

Bashkatov, A., E. Genina, et al. (2005). "Optical properties of human skin, subcutaneous and mucous tissues in the wavelength range from 400 to 2000 nm." Journal of Physics D: Applied Physics **38**: 2543.

Bashkatov, A., E. Genina, et al. (2005). "Optical properties of the subcutaneous adipose tissue in the spectral range 400–2500 nm." Optics and spectroscopy **99**(5): 836-842.

Benaron, D. A., I. H. Parachikov, et al. (2005). "Design of a visible-light spectroscopy clinical tissue oximeter." Journal of Biomedical Optics **10**: 044005.

Bigio, I. J., S. G. Bown, et al. (2000). "Diagnosis of breast cancer using elastic-scattering spectroscopy: preliminary clinical results." Journal of Biomedical Optics **5**: 221.

Brynolf, M., Sommer, M., Desjardins, A.E., et al. "Optical Detection of the Brachial Plexus for Peripheral Nerve Blocks: an in-vivo swine study." submitted to Regional Anesthesia and Pain Medicine.

Bu, S., S. Xie, et al. (2008). "Short communication: The preliminary research of identifying motor and sensory fascicles using near infrared diffuse reflectance spectroscopy in combination with soft independent modelling of class analogy." Journal of Near Infrared Spectroscopy **16**: 431.

Curatolo, M., A. Orlando, et al. (1995). "A multifactorial analysis to explain inadequate surgical analgesia after extradural block." Br J Anaesth **75**(3): 274-281.

Desjardins, A. E., Hendriks, B.H.W., van der Voort, M., Nachabe, R. "Epidural needle with embedded optical fibers for spectroscopic differentiation of tissue: ex vivo feasibility study." submitted to Biomedical Optics Express

Desjardins, A. E., van der Voort, M., Roggeveen, S., Lucassen, G.W., Bierhoff, W., Hendriks, B.H.W. "Needle Stylet with Integrated Optical Fibers for Spectroscopic Contrast during Peripheral Nerve Blocks." submitted to Journal of Biomedical Optics.

Fredman, B., M. B. Nun, et al. (1999). "Epidural Steroids for Treating “Failed Back Surgery Syndrome”: Is Fluoroscopy Really Necessary?" Anesthesia & Analgesia **88**(2): 367.

Kondepati, V. R., H. M. Heise, et al. (2008). "Recent applications of near-infrared spectroscopy in cancer diagnosis and therapy." Analytical and Bioanalytical Chemistry **390**(1): 125-139.

Liese, G. J., W. Pong, et al. (1985). "Fiber-optic stylet for needle tip localization." Appl. Opt **24**: 3125-3127.

Loubert, C., S. R. Williams, et al. (2008). "Complication during ultrasound-guided regional block: accidental intravascular injection of local anesthetic." Anesthesiology **108**(4): 759.

Lubawy, C. and N. Ramanujam (2004). "Endoscopically compatible near-infrared photon migration probe." Optics letters **29**(17): 2022-2024.

McLeod, A., A. Roche, et al. (2005). "Case series: Ultrasonography may assist epidural insertion in scoliosis patients." Can J Anaesth **52**(7): 717-720.

Nachabé, R., B. H. W. Hendriks, et al. (2010). "Estimation of lipid and water concentrations in scattering media with diffuse optical spectroscopy from 900 to 1600 nm." Journal of Biomedical Optics **15**: 037015.

Nachabé, R., B. H. W. Hendriks, et al. (2010). "Estimation of biological chromophores using diffuse optical spectroscopy: benefit of extending the UV-VIS wavelength range to include 1000 to 1600 nm." Biomedical Optics Express **1**(5): 1432-1442.

Nezhat, C., M. K. Terris, et al. (2004). "Continuous, noninvasive, and localized microvascular tissue oximetry using visible light spectroscopy." Anesthesiology **100**(6): 1469.

Radhakrishnan, H., A. Senapati, et al. (2005). "Light scattering from rat nervous system measured intraoperatively by near-infrared reflectance spectroscopy." Journal of Biomedical Optics **10**: 051405.

Radhakrishnan, H., A. Senapati, et al. (2005). Near-infrared reflectance spectroscopy as a novel method to detect demyelination in rat sciatic nerve in vivo.

Rathmell, J. P. (2005). Atlas of image-guided intervention in regional anesthesia and pain medicine, Lippincott Williams & Wilkins.

Rathmell, J. P., A. E. Desjardins, et al. (2010). "Identification of the Epidural Space with Optical Spectroscopy: An In Vivo Swine Study." Anesthesiology **113**(6): 1406.

Rigg, J. R., K. Jamrozik, et al. (2002). "Epidural anaesthesia and analgesia and outcome of major surgery: a randomised trial." Lancet **359**(9314): 1276-1282.

Rithalia, S. V. S. (1991). "Developments in transcutaneous blood gas monitoring: a review." Journal of medical engineering & technology **15**(4-5): 143-153.

Schenkman, K. A., D. R. Marble, et al. (1999). "Optical spectroscopic method for in vivo measurement of cardiac myoglobin oxygen saturation." Applied spectroscopy **53**(3): 332-338.

Ting, C. K., M. Y. Tsou, et al. (2010). "A new technique to assist epidural needle placement: Fiberoptic-guided insertion using two wavelengths." Anesthesiology **112**(5): 1128.

VadeBoncouer, T. R., G. L. Weinberg, et al. (2008). "Early detection of intravascular injection during ultrasound-guided supraclavicular brachial plexus block." Regional Anesthesia and Pain Medicine **33**(3): 278.

van Kleef, M. (2010). "Protocol NL31578.068.10/MEC 09-1-075 ”Optical Tissue Stylet – observational study in humans”."

van Veen, R. L. P., A. Amelink, et al. (2005). "Optical biopsy of breast tissue using differential path-length spectroscopy." Physics in medicine and biology **50**: 2573.

Voort, M. v. d. (2011). "PhN-11043 Risk analysis first generation."

Xie, S., B. R. Xiang, et al. (2009). "Rapid identification of anterior and posterior root of cauda equina nerves by near-infrared diffuse reflectance spectroscopy." Journal of Biomedical Optics **14**: 024005.

Zetlaoui, P. J., J. P. Labbe, et al. (2008). "Ultrasound guidance for axillary plexus block does not prevent intravascular injection." Anesthesiology **108**(4): 761.

Zonios, G., L. T. Perelman, et al. (1999). "Diffuse Reflectance Spectroscopy of Human Adenomatous Colon Polyps< i> In Vivo</i>." Applied Optics **38**(31): 6628-6637.

APPENDIX 2 – Proefpersoon informatie

See separate document ("INFORMATIE VOOR VRIJWILLIGERS Optische weefsel canule – observationele studie naar het aanprikken van bloedvaten in mensen", version 1.0, 31 Maart 2011).

APPENDIX 3 – Serious Adverse Event Report Form

Appendix 3
© Philips RESEARCH

**PHILIPS RESEARCH Q&R office**	**Serious Adverse Event Report Form**
	Study title: Optical Tissue Stylet – observational study into vascular access in humans

The report form MUST be received at Philips Research Q&R Office within 1 business day of when you first learn about the incident!

Complete form and send it via one of the following methods:

Email: QandRoffice.pre @philips.com (preferred method)
or
FAX: Philips Research Q&R Office +31 40 274 6321

As an additional measure we ask you to notify the Philips Research Q&R office by telephone whenever an e-mail or fax has been sent.
Phone number: +31 40 274 0430

PRINT LEGIBLE!

* **Required Information @ Submission.**

**(A) REPORTER'S INFORMATION (person completing this form)**	
***1. Name**	
***2. Title**	
***3. Institution Name**	
***4. Mailing Address**	
***5. Postal Code**	
***6.City**	
***7. Email address**	
***8. Telephone**	**country code: + city code: -**
**(B) PRINCIPAL INVESTIGATOR'S INFORMATION (if not the reporter)**	
***1. Name**	
***2. Title**	
***3. Institution Name**	
***4. Mailing Address**	
***5. Postal Code**	
***6.City**	
***7. Email address**	
***8. Telephone**	**country code: + city code: -**
**(C) DATES [use dd/mmm/yyyy format** (e.g. 10/AUG/2010)**]**	
***1. Date and time of incident**	
***2. Initiation Date** (date that the reporter was informed about the incident?)	

The information contained herein has not been sustained and may be inaccurate or incomplete. As such, the information does not constitute an admission or confirmation by the individual reporting, that the device actually malfunctioned, caused or contributed to a death or serious injury defined by the regulatory agencies.

Appendix 3
© Philips RESEARCH

**PHILIPS RESEARCH Q&R office**	**Serious Adverse Event Report Form**
	Study title: Optical Tissue Stylet – observational study into vascular access in humans

**(D) STUDY SUBJECT RELATED INFORMATION**	
***1. Subject ID**	
2. Provide sex and age.	
3. Describe the condition being treated and status of the subject before the alleged incident	
**(E) DEVICE (include ALL devices linked to alleged incident)**	
***1. Device/System name**	
2. Model # /System #	
3. Serial #	
4. Lot #, Date Code, Software Rev.	
5. Does the user / patient / customer allege that the device used in the contributed to the incident? If so, how? (Identify who made the statement , i.e. Doctor, Nurse, etc.)	
**(F) INCIDENT DESCRIPTION**	
***1. Brief description of the nature of the incident (attach description if more space is needed)**	
***2. Category of the incident**	☐ **Death**<br>☐ **Life threatening**<br>☐ **Hospitalization – initial or prolonged**<br>☐ **Disability**<br>☐ **Required intervention to prevent permanent impairment**<br>☐ **Other:**<br>……………………………………………………………….
3.If additional information is needed, who should be contacted by Philips?	Identify contact and provide name.
**(G) NOTIFICATION/ RESPONSE**	
1. Has anyone else been notified?	Yes/No/Unknown. If Yes, provide name:
2. Has this incident been reported to the Competent Authority?	Yes/No; If Yes provide report date and regulatory file #, if known:

The information contained herein has not been sustained and may be inaccurate or incomplete. As such, the information does not constitute an admission or confirmation by the individual reporting, that the device actually malfunctioned, caused or contributed to a death or serious injury defined by the regulatory agencies.

Appendix 3
© Philips RESEARCH

**PHILIPS RESEARCH Q&R office**	**Serious Adverse Event Report Form**<br>Study title: Optical Tissue Stylet – observational study into vascular access in humans

**(H) ADDITIONAL INFORMATION, IF ANY**
**(I) SIGNATURE**
Signature of Principle Investigator or Reporter

The information contained herein has not been sustained and may be inaccurate or incomplete. As such, the information does not constitute an admission or confirmation by the individual reporting, that the device actually malfunctioned, caused or contributed to a death or serious injury defined by the regulatory agencies.
