# Supplementary material for: Spectral tissue sensing to identify intra- and extravascular needle placement — A randomized single-blind controlled trial
Source: PLoS One. 2017 Mar 9;12(3):e0172662. doi: 10.1371/journal.pone.0172662 (PMC5344374; doi:10.1371/journal.pone.0172662)
Supplement: S2 File — (PDF) [file pone.0172662.s002.pdf]

UMC St Radboud

Instituut Waarborging Kwaliteit en Veiligheid
Commissie Mensgebonden Onderzoek

Huispost 578
Postbus 9101
6500 HB Nijmegen

UMC St Radboud Centraal, route 578
Geert Grooteplein 10

T (024) 361 31 54

cmo@iwkv.umcn.nl
www.umcn.nl
www.cmoregio-a-n.nl

578

Drs. I.J. Bruaset,
Sint Maartenskliniek,
Afdeling Anesthesiologie,
Huispost 4612

Ons Kenmerk
MvdV/CMO 0742

Datum
29 juni 2011

Titel: Optical Tissue Stylet – oberservational study into vascular access in humans
Registratienummer: 2011/198
NL nr.: NL 36528.091.11

Geachte heer Bruaset,

Bijgevoegd treft u aan het positieve oordeel van de CMO Regio Arnhem-Nijmegen over bovengenoemd onderzoek.

Dit betekent dat het onderzoek kan worden uitgevoerd in het centrum dat in het positieve oordeel wordt genoemd nadat de Raad van Bestuur/Directie van het centrum daarvoor toestemming heeft verleend.

Indien het voornemen bestaat het onderzoek ook nog in een ander centrum uit te voeren, dan dient aan de CMO Regio Arnhem – Nijmegen een positieve verklaring van de Raad van Bestuur/Directie van het betreffende centrum over de lokale uitvoerbaarheid van het onderzoek te worden overlegd. De CMO Regio Arnhem-Nijmegen kan vervolgens het positieve oordeel uitbreiden naar het betreffende centrum.

Bij correspondentie over het onderzoek gelieve titel en registratienummer te vermelden.

Ik vertrouw erop u met dit schrijven van dienst te zijn en namens de commissie wens ik u succes met de uitvoering van het onderzoek.

Met vriendelijke groet,
Namens de CMO Regio Arnhem-Nijmegen

Mr. dr. F.M. van Agt

Cc: - CCMO (digitaal)

UMC St Radboud

BESLUIT betreffende onderzoek:
Titel: Optical Tissue Stylet – oberservational study into vascular access in humans
Registratienummer: 2011/198
NL nr.: NL 36528.091.11

Contactpersoon: Drs. I.J. Bruaset, Sint Maartenskliniek, Afdeling Anesthesiologie, Huispost 4612
Verrichter: Philips

Bevoegdheid
De Commissie Mensgebonden Onderzoek (CMO) Regio Arnhem – Nijmegen heeft, gelet op het bepaalde in artikel 2, tweede lid, onder a van de Wet medisch-wetenschappelijk onderzoek met mensen (WMO), kennis genomen van bovengenoemd onderzoek, dat door haar op **5 mei 2011** ter beoordeling werd ontvangen.

Besluit
De CMO Regio Arnhem - Nijmegen oordeelt positief over het onderzoek, zoals beschreven in de volgende documenten:

A	Aanbiedingsbrief van drs. I.J. Bruaset d.d. 4 mei 2011
	Machtiging van Philips d.d. 1 april 2011
	Brief van drs. I.J. Bruaset d.d. 16 juni 2011
B	ABR-formulier versie 03 d.d. 10 juni 2011
C	Onderzoeksprotocol versie 2.0 d.d. 6 juni 2011
E	Patiënteninformatie versie 1.0 d.d. 31 maart 2011
	Toestemmingsformulier (ongedateerd)
	Advertentietekst (ongedateerd)
G	Aansprakelijkheidsverzekeringscertificaat AON d.d. 8 juni 2011
	Proefpersonen verzekeringscertificaat AON d.d. 8 juni 2011
H	CV onafhankelijk arts
I	Verklaring afdelingshoofd (BROK)
	CV hoofdonderzoeker Prof. dr. G.J. Scheffer
K	Onderzoekscontract (A11-0246) d.d. 24 mei 2011
	Verklaring van de instrumentele dienst d.d. 20 juni 2011
	Declaration of Conformity PhotonicNeedle optical console for 'OTS VA' study

Dit positieve oordeel geldt voor het volgende centrum:

Centrumnaam	Onderzoekers/Onderzoeksleiders
UMC St Radboud	Prof. dr. G.J. Scheffer

Motivering
Bij haar oordeelsvorming heeft de CMO Regio Arnhem-Nijmegen de bij en krachtens de WMO geldende en van toepassing zijnde bepalingen in acht genomen. Daarnaast heeft de CMO Regio Arnhem-Nijmegen bij haar oordeelsvorming onder meer de bepalingen uit de ICH/GCP-richtsnoer (voor zover relevant) betrokken.

UMC St Radboud

De CMO Arnhem – Nijmegen is van oordeel dat:

- aannemelijk is dat het onderzoek zal resulteren in een nieuw inzicht op het gebied van de medische wetenschappen;
- het nieuwe inzicht niet verkregen kan worden op een wijze die minder bezwaarlijk is uit het oogpunt van de bescherming van proefpersonen;
- het belang van het nieuwe inzicht opweegt tegen de bezwaren die voor proefpersonen verbonden zijn aan deelname aan het onderzoek;
- de informed consent-procedure zorgvuldig is vormgegeven;
- het onderzoek ook overigens voldoet aan redelijkerwijs daaraan te stellen en toetsbare eisen.

Verzekering
De CMO Regio Arnhem – Nijmegen heeft vastgesteld dat op correcte wijze uitvoering is gegeven aan de verzekeringsplicht van artikel 7 WMO, zoals uitgewerkt in het Tijdelijk besluit verplichte verzekering bij medisch-wetenschappelijk onderzoek met mensen.

**Proefpersonen uit**	**Vallen onder de WMO-verzekering die is afgesloten door**
UMC St Radboud	Philips

De CMO Regio Arnhem – Nijmegen heeft voorts vastgesteld dat op correcte wijze uitvoering is gegeven aan de in WMO artikel 7 lid 6 genoemde aansprakelijkheid.

**Centrum**	**Valt onder de aansprakelijkheidsverzekering die is afgesloten door**
UMC St Radboud	Philips

Beoordelingsprocedure
Bij de beoordeling van het onderzoek is door de CMO Regio Arnhem Nijmegen de procedure gevolgd die geregeld is in de artikelen 17 t/m 21 van haar reglement. Het reglement is te vinden op de website van de CMO Regio Arnhem – Nijmegen (www.cmoregio-a-n.nl).

Verplichtingen
- De CMO Regio Arnhem – Nijmegen moet op de hoogte worden gebracht van de startdatum van het onderzoek. Dat is de datum waarop begonnen wordt met de werving van (de eerste) proefperso(o)n(en) in Nederland. Het positieve oordeel verliest zijn geldigheid als met het uitvoeren van het onderzoek niet is begonnen binnen een jaar nadat dit besluit genomen is.
- Amendementen van het onderzoek dienen *met redenen omkleed* ter beoordeling bij de CMO Regio Arnhem – Nijmegen ingediend te worden. Daarbij dient steeds expliciet aangegeven te worden welke gevolgen het amendement heeft voor de proefpersonen.
- Met inachtneming van het bepaalde in artikel 10 WMO moet de CMO Regio Arnhem – Nijmegen om een nader oordeel worden verzocht als het onderzoek een verloop neemt dat in noemenswaardige mate voor de proefpersoon ongunstiger is dan in het protocol is voorzien. De indiening moet vergezeld te gaan van een zelfstandige beoordeling over het (mogelijk) causale verband tussen de nadelige gebeurtenis en de deelname aan het onderzoek, alsmede over het gevolg dat nadelige gebeurtenis dient te hebben voor de voortzetting (al dan niet in gewijzigde vorm) van het onderzoek.
- Met inachtneming van het bepaalde in de artikelen 13p en 13q van de WMO en het gestelde op p.47 en 49 van de Instruction Manual (Ministerie van VWS d.d. oktober 2005) moeten vermoedens van ernstige bijwerkingen (SUSARS en SSARS) ter beoordeling aan de CMO Regio Arnhem-Nijmegen te worden ingediend (raadpleeg de website van de commissie voor de te volgen procedure).
- De voortijdige beëindiging van het onderzoek moet binnen 15 dagen aan de CMO Regio Arnhem – Nijmegen gemeld worden. Hierbij moet worden aangegeven wat de reden is voor de voortijdige beëindiging.

- Duurt het onderzoek langer dan gepland, dan moet dit terstond aan de CMO Regio Arnhem – Nijmegen gemeld te worden. Hierbij moet worden aangegeven wat de reden is voor de overschrijding van de geplande duur.
- De afronding van het onderzoek, dat wil zeggen de datum waarop de laatste proefpersoon in Nederland de laatste controle in het kader van het onderzoek heeft afgelegd, moet binnen 3 maanden aan de commissie gemeld worden.
- De resultaten van het onderzoek moeten aan de CMO Regio Arnhem Nijmegen gemeld worden, in de vorm van een (voorgenomen of *gesubmitte*) publicatie. Worden de resultaten niet ter publicatie aangeboden dan moet dit onder vermelding van de reden aan de commissie gemeld worden.

Administratief beroep
Degene wiens belang rechtstreeks bij dit besluit betrokken is, kan op grond van artikel 23 van de WMO tegen het besluit binnen zes weken na de dag waarop dit besluit bekend is gemaakt een administratief beroepschrift indienen bij de Centrale Commissie Mensgebonden Onderzoek (CCMO). Het beroepschrift moet worden geadresseerd aan: CCMO, Postbus 16302, 2500 BH Den Haag.

Hoogachtend,
Namens de CMO Regio Arnhem – Nijmegen
Dr. F.Th.M. Huysmans, voorzitter

Voor deze,

Mr. dr. F.M. van Agt

Nijmegen, 29 juni 2011

Leden
Dr. F. Huysmans, nefroloog, voorzitter
Dr. M.J.J. Prick, neuroloog, vice-voorzitter
Dr. P.G.M. van der Valk, dermatoloog, vice-voorzitter
Mr. Dr. F.M. van Agt, jurist
Ir. Th. de Boo, methodoloog
farmacoloog
Mw. A.J.F.H. Bossmann, verpleegkundige
Mw. E.P. Bunt, "lekenlid"
Mw. Mr. W.G.H. Corté, jurist
Dr. J. Deinum, internist
Mw. W.J. Deurman-Peters, "lekenlid"
Mw. Dr. C.A. Eggink, oogarts
Mw. H.Th.M. van der Eng, verpleegkundige
Drs. A.B. Feuth, methodoloog
Mw. Dr. P.M.G. Filius, ziekenhuisapotheker
Dr. Q. van Hoesel, oncoloog
Mw. Mr. M.S. Janssen, juriste
Mw. Mr. K.W.J. Koller, "lekenlid"
Prof. Dr. F. Kraaimaat, psycholoog
Dr. C. Kramers, internist/klinisch farmacoloog
Mw. Dr. A. Lelie, ethica
Drs. S.J. van Leeuwen, intensivist
Mw. Dr. D.M.W.M. te Loo, kinderarts
Prof. dr. L.F.A.G. Massuger, gynaecoloog
Mw. Dr. S.S. Natsch, ziekenhuisapotheker/klinisch

Dr. M.A.M. Pijnenburg, ethicus
Dr. G.A.M. Pop, cardioloog
Dr. G. Rongen, internist/klinisch farmacoloog
Mw. Dr. M.M. Roovers, ethica
Dr. A. Schattenberg, internist/hematoloog
Dr. N. Steinkamp, ethicus
Dr. I. Tendolkar, psychiater
Mw. Mr. F.E.A.M. Tesser, jurist
Dr. B.P.A. Thoonen, huisarts
Mw. M. Verstappen, verpleegkundige
Dr. H. Visser, reumatoloog
Drs. J.C. van der Vorm, ethicus
Dr. W.N.P. Willemsen, gynaecoloog

Adviseurs
Dr. J.W. Mouton, arts, medisch microbioloog
Dr. Ir. W.K.J. Renema, klinisch fysicus
