# Supplementary material for: Spectral tissue sensing to identify intra- and extravascular needle placement — A randomized single-blind controlled trial
Source: PLoS One. 2017 Mar 9;12(3):e0172662. doi: 10.1371/journal.pone.0172662 (PMC5344374; doi:10.1371/journal.pone.0172662)
Supplement: S1 Table — Individual 14 was excluded. Number 1 means first position, number 2 second position. Intended subcutaneous position (S), intended vasculair needle position (V). More data (ultrasound images, optical data and CRFs) could be found on figshare.com (project name: Spectral tissue sensing to identify intra-and extravascular needle placement). (PDF) [file pone.0172662.s004.pdf]

Volunteer	Location	Position	B
1	1	S	2
1	2	V	1501
2	1	S	1
2	2	S	1
3	1	S	0
3	2	S	5
4	1	S	3
4	2	V	28586
5	1	S	5
5	2	S	12
6	1	S	4
6	2	V	1416
7	1	S	2
7	2	V	382
8	1	S	2
8	2	S	2
9	1	S	2
9	2	S	1
10	1	S	1
10	2	S	8
11	1	S	2
11	2	S	8
12	1	S	2
12	2	V	1664
13	1	S	0
13	2	V	2
15	1	S	2
15	2	S	4
16	1	S	0
16	2	V	2915
17	1	S	0
17	2	S	0
18	1	S	3
18	2	S	1
19	1	S	1
19	2	V	1
20	1	S	1
20	2	S	1
